# Does conflict help or hurt cognitive control? Initial evidence for an inverted U-shape relationship between perceived task difficulty and conflict adaptation

**DOI:** 10.3389/fpsyg.2015.00974

**Published:** 2015-07-10

**Authors:** Henk van Steenbergen, Guido P. H. Band, Bernhard Hommel

**Affiliations:** Leiden Institute for Brain and Cognition and Institute of Psychology, Cognitive Psychology Unit, Leiden UniversityLeiden, Netherlands

**Keywords:** cognitive control, motivation, task difficulty, effort, pupil dilation

## Abstract

Sequential modulation of congruency effects in conflict tasks indicates that cognitive control quickly adapts to changing task demands. We investigated in four experiments how this behavioral congruency-sequence effect relates to different levels of perceived task difficulty in a flanker and a Stroop task. In addition, online measures of pupil diameter were used as a physiological index of effort mobilization. Consistent with motivational accounts predicting that increased levels of perceived task difficulty will increase effort mobilization only up to a certain limit, reliable dynamic conflict-driven adjustment in cognitive control was only observed when task difficulty was relatively low. Instead, tasks tentatively associated with high levels of difficulty showed no or reversed conflict adaptation. Although the effects could not be linked consistently to effects in self-reported task difficulty in all experiments, regression analyses showed associations between perceived task difficulty and conflict adaptation in some of the experiments, which provides some initial evidence for an inverted U-shape relationship between perceived difficulty and adaptations in cognitive control. Furthermore, high levels of task difficulty were associated with a conflict-driven reduction in pupil dilation, suggesting that pupil dilation can be used as a physiological marker of mental overload. Our findings underscore the importance of developing models that are grounded in motivational accounts of cognitive control.

## Introduction

In a constantly changing environment, cognitive control helps to adaptively respond to task demands. Paradigms such as the flanker task (Eriksen and Eriksen, 1974) and the Stroop task (Stroop, 1992) have been designed to probe cognitive control processes. In the flanker task, for example, people respond to a central target while ignoring flanking distracters. The reaction-time difference between trials with target-congruent and -incongruent flankers has been called the congruency effect and can be used as a measure of sustained cognitive control. On the other hand, dynamic adjustments in control are reflected in trial-to-trial adaptations. This sequential effect typically indicates that the congruency effect on the current trial is reduced when it follows an incongruent as compared to a congruent trial (Greenwald and Rosenberg, 1978; Gratton et al., 1992).

Conflict monitoring theory assumes that congruency-sequence effects occur because incongruent trials evoke response conflict, which triggers control improvements and thus reduces interference on subsequent trials (Botvinick et al., 2001; Duthoo et al., 2014). Indeed, numerous studies have found evidence for this so-called conflict-adaptation effect. Conflict adaptation has been demonstrated across several conflict paradigms such as the flanker, Stroop, and Simon tasks (Egner, 2007; Duthoo et al., 2014). Accumulating neuroimaging research has identified a possible neural mechanism that involves a conflict monitoring system, mediated by regions in the medial prefrontal cortex, that helps to adapt control through the enhancement of task-goal representations in more lateral prefrontal areas (Kerns et al., 2004; Egner and Hirsch, 2005a).

Although the congruency-sequence effect typically is investigated in the context of the conflict monitoring theory (Botvinick et al., 2001), the adaptation observed might actually be driven by a more general motivational mechanism that could be described with the seminal ‘difficulty law of motivation’ introduced by Ach and co-workers (Hillgruber, 1912; Ach, 1935). According to this motivational account, increases in task difficulty motivate organisms to mobilize additional effort. Mirroring physical effort mobilization, the amount of mental effort invested in the task is proportional to the level of perceived task difficulty. However, effort is withdrawn as soon as success is perceived as no longer possible or worthwhile (Kahneman, 1973; Brehm and Self, 1989; Tops et al., 2015), resulting in an inverted U-shape relationship between task difficulty and effort. In the current study we investigate the hypothesis that improved goal-directed behavior as measured by typical sequential effects in conflict tasks likewise follows an inverted U-shape relationship.

Although the majority of the available studies that have tested the motivational account of effort mobilization have only provided physiological and self-report evidence (for reviews, see Wright and Kirby, 2001; Gendolla et al., 2011), a recent study by Dreisbach and Fischer (2011) has shown that adjustments in effort mobilization can also be observed in a behavioral paradigm through sequential effects. In that study, sequence effects in reaction times (RTs) were observed in a perceptual fluency tasks using different levels of task difficulty, demonstrating that sequential behavioral adaptation can occur even in the absence of conflict.

The aim of the current study is to investigate how behavioral congruency-sequence effects in conflict tasks interact with different levels of perceived task difficulty. As outlined above, conflict-driven improvement of control is likely to reflect a momentary increase in effort driven by the difficulty of the previous trial; the goal of the current investigation is to understand whether conflict-driven control also occurs under conditions where difficulty levels further increase. Motivational accounts of effort mobilization have suggested that difficulty will only increase effort mobilization up to some upper limit, after which it may reach asymptote or drops (Kahneman, 1973; Brehm and Self, 1989). Likewise, dynamic conflict-driven increases in effort may only occur if trial difficulty does not exceed this critical threshold. If this is true, we would predict that conflict adaptation only occurs in cases where sustained task difficulty is not too high. The present study puts this prediction to an empirical test.

A first indication that congruency-sequence effects indeed become smaller under conditions in which the execution of the primary task is more difficult comes from studies that have analyzed sequential adaptation in the context of dual-task situations. Comparing Simon-task performance under single and dual-task situations, Sturmer et al. (2005) observed smaller congruency-sequence effects in a dual-task context, indicating that the secondary task may have consumed resources needed for conflict-driven improvements in control. In another study by Fischer et al. (2008), processing demands and response conflict were manipulated within the same trial, using a numerical judgment task in the context of a Simon paradigm. Consistent with a limited resources account, difficult number judgments reduced the subsequent congruency-sequence effect in Simon performance (but cf. Fischer et al., 2010).

The present study aims to find evidence for task-difficulty effects on behavioral adaptations in a series of four experiments. In Experiments 1 and 2, we compared how differences in the size of the conflict-adaptation effect in a Stroop and a flanker task may be related to differences in perceived task difficulty. However, given that it is difficult to draw firm conclusions from a comparison of two different types of tasks (see later Discussion), in Experiment 3 we aimed to causally manipulate task difficulty using task instruction that emphasized speed while maintaining accuracy. Finally, in Experiment 4 we tested a large sample of participants in order to demonstrate that individual differences in perceived difficulty of incongruent trials within a given task can predict the size of the adaptation effect. Our study provides some initial evidence for an inverted U-shape relationship between perceived task difficulty and conflict adaptation, suggesting that when difficulty is high, congruency-sequence effects are small, whereas when difficulty is low, perceived difficulty predicts increased conflict adaptation.

## Experiment 1: Re-analysis of van Steenbergen et al. (2010)

In a first attempt to test whether differences in task difficulty can account for differences in conflict-adaptation effects, we re-analyzed an earlier published data set (van Steenbergen et al., 2010) by comparing congruency-sequence effects as a function of the level of task demands participants reported. We predicted that improvements in cognitive control driven by previous-trial conflict were absent under conditions of high task difficulty. That is, we predicted that a task that is associated with overall high task demands may show smaller congruency-sequence effects.

### Methods

For detailed methods, see van Steenbergen et al. (2010).

#### Participants

Ninety-eight students participated either for payment or course credits (18–30 years old; 24 males; 11 left-handed). Data from seven participants were excluded from analyses because of response omissions on more than 20% of the trials (2), chance level task performance (3), or incompliance with instructions (2). Data were pooled across four different mood induction groups, as the mood conditions were irrelevant for the purpose of the current study.

#### Tasks

Two variants of a classic cognitive-control paradigm were used to measure conflict adaptation. An adapted version of the flanker task (Eriksen and Eriksen, 1974) consisted of centrally presented target stimuli which were vertically flanked on either side by two identical response-congruent or response-incongruent stimuli. Stimulus-incongruent combinations of targets and distractors associated with congruent responses were not presented. An adapted version of the Stroop task (Stroop, 1992) consisted of a column of five identical stimuli likewise presented in response-congruent or response-incongruent ink colors. Flanker and Stroop stimuli were carefully matched by using sets of Dutch color words. Each task used a counterbalanced unique set of four words. Two of these stimuli were mapped to a left hand response, and the other two stimuli were mapped to a right hand response.

E-Prime software was used for stimulus presentation and response recording. All trials started with a fixation cross (randomly varying intervals were set to 800, 1000, or 1100 ms), followed by the stimulus, which was presented until response registration or, in the case of omission, for 1500 ms. In half of the trials the stimuli would call for different responses (Incongruent [I] condition; e.g., the word “green” surrounded by the words “yellow” in the flanker task and the word “blue” printed in red in the Stroop task) whereas in the other half identical target and distracter dimensions would call for the same response (Congruent [C] condition; e.g., the word “green” surrounded by the words “green” in the flanker task and the word “blue” printed in blue in the Stroop task). All trials were presented in an unconstrained random sequence. Stimuli appeared in lower-case in Arial bold font (3.5 cm wide and 5.4 cm high) and were presented on a gray background. Flanker stimuli used black ink color. Participants viewed the stimuli on a 17″ monitor from about 60 cm.

#### Procedure

Instructions emphasized both speed and accuracy. Following 16 practice trials, and a 10-min mood induction, participants performed a flanker and a Stroop task block (in counterbalanced order), which were repeated after a short 3-min mood booster. A textual reminder of the stimulus-response mapping was shown for 15 s before the start of each of the four blocks of 72 trials. At the end of the experiment, participants evaluated the flanker and Stroop task in terms of weariness, unpleasantness and difficulty on a 6-points scale.

### Results

#### Subjective Reports

Task difficulty ratings showed that the Stroop task was associated with higher demands than the flanker task [4.1 versus 3.7; *t*(90) = 2.6, *p* < 0.05]. Weariness and unpleasantness scores were not different for the tasks [*t*(90)s < 1.6, *p*s > 0.12].

#### Behavioral Results

**FIGURE 1:**
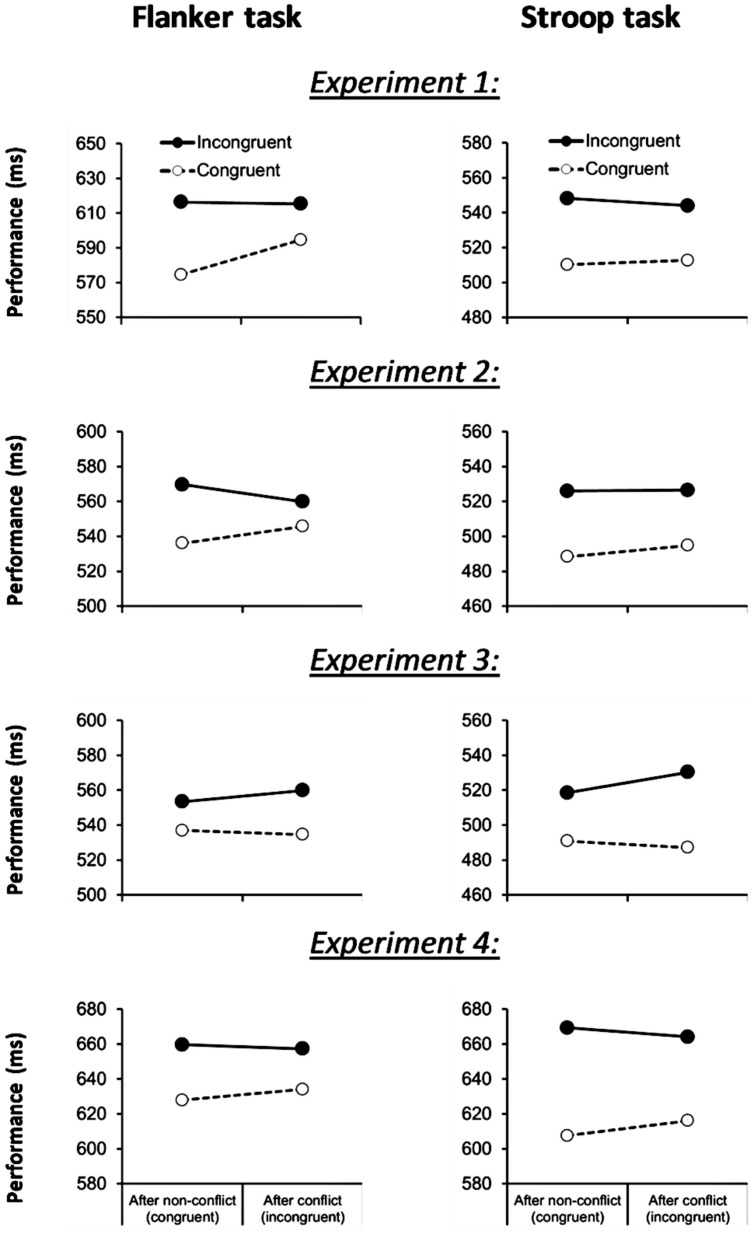
**Flanker task (left column) and Stroop task (right column) performance across Experiment 1, 2, 3, and 4, as a function of current-trial congruency and previous-trial congruency**.

The first trial of each block (1.4%) and trials not complying with the outlier criterion (2 SDs; 4.7%) were excluded from all analyses. We first ran analyses on the two tasks separately. ANOVAs on correct RT data revealed significant basic congruency effects for both the flanker task [31 ms; *F*(1,90) = 137.9, *p* < 0.001] and the Stroop task [35 ms; *F*(1,90) = 71.9, *p* < 0.001] confirming that both paradigms can reliably measure cognitive control. However, as **Figure 1** shows, a congruency-sequence effect, i.e., a reduction of the congruency effect following conflict, was only found for the flanker task [21 ms; *F*(1,90) = 17.2, *p* < 0.001] but not for the (more difficult) Stroop task [7 ms; *F*(1,90) = 1.4, *p* > 0.2]. Accuracy data confirmed the basic congruency effects for the flanker task [2.5%; *F*(1,90) = 22.3, *p* < 0.001] and the Stroop task [2.5%; *F*(1,90) = 18.5, *p* < 0.001]. There was a trend for a congruency-sequence effect in the flanker task [2.0%; *F*(1,90) = 3.68, *p =* 0.058]. In addition to the congruency-sequence effect in the flanker task, participants slowed down their response following conflict [*F*(1,90) = 11.4, *p* < 0.005] whereas accuracy was not affected (*F* < 1; cf. Ullsperger et al., 2005).

An additional ANOVA was run to statistically compare the effects of task directly. There was a trend for an interaction between task and the congruency-sequence effect in RT, *F*(1,90) = 3.3, *p* = 0.073, MSE = 707.8, but not in accuracy, *F* = 0.2. There was also a main effect of task on RT indicating faster responses on the Stroop task (529 ms) than the flanker task (600 ms), *F*(1,90) = 100.4, *p* < 0.001, MSE = 9241.3, and a significant interaction between task and previous-trial congruency on RT, *F*(1,90) = 7.6, *p* = 0.007, MSE = 640.5.

#### Relationship between Perceived Task Difficulty and Conflict Adaptation

In order to investigate whether individual differences in perceived task difficulty predicted the size of the conflict adaptation effects, we regressed individual task difficulty ratings on individual scores of conflict adaptation (calculated as the reduction of the congruency effect following conflict versus non-conflict trials in RT), separately for the flanker task and the Stroop task, using linear and quadratic curve fitting as implemented in SPSS. No reliable associations were observed (*F*s < 1).

### Discussion

Experiment 1 provides some preliminary support for the hypothesis that task difficulty may be an important factor that accounts for reduced congruency-sequence effects: a Stroop task that was reported to be more demanding yields less conflict adaptation than a version of the flanker task. That is, while a reliable congruency-sequence effect was observed in the flanker task, the much smaller effect observed in the Stroop task was not statistically significant, despite the large sample (*N* = 91).

## Experiment 2

Experiment 1 provided limited initial evidence in favor of the hypothesis that high overall task difficulty might reduce conflict adaptation. However, these data were pooled over several mood induction groups which produced different adaptation effects in the earlier published study (van Steenbergen et al., 2010). It might thus be argued that the overall size of conflict-adaptation effects cannot be generalized to the affectively more neutral situations commonly used in laboratory studies. This motivated us to design a replication study.

In the follow-up study we also recorded pupillary dilation to provide a measure of effort mobilization (Hess and Polt, 1964; Kahneman, 1973). Although pupil size is also determined by other variables, it has been repeatedly shown that task-related pupil dilation systematically increases as a function of task difficulty or processing load and thus “provides a powerful analytic tool for the experimental study of processing load and the structure of processing resources” (Beatty, 1982, p. 291; Beatty and Lucero-Wagoner, 2000). Interestingly, consistent with motivational accounts of effort mobilization (Kahneman, 1973; Brehm and Self, 1989; Tops et al., 2015), when cognitive resources can no longer meet the effort required for the task, no further dilation occurs and dilation either reaches asymptotic value or declines (Poock, 1973; Peavler, 1974; Granholm and Steinhauer, 2004; Cabestrero et al., 2009). Other measures of arousal show a similar pattern of responding (Pavlov, 1955; Tops et al., 2015). The decline in pupil diameter under conditions of mental overload exclusively occurs when people keep trying to work on the task (Granholm et al., 1996).

In the context of cognitive control tasks, numerous pupillometry studies have already shown that incongruent trials in the Stroop task (Brown et al., 1999; Siegle et al., 2004; Laeng et al., 2011), the flanker task (van Bochove et al., 2013; Wendt et al., 2014), and the Simon task (van Steenbergen and Band, 2013) increase pupil dilation. This finding is consistent with motivational accounts that suggest the operation of an energy-saving motivational process in which additional effort is recruited to meet the increased demands imposed by conflict trials in these paradigms (cf. Hull, 1943; de Galan et al., 2014).

It is thus possible that trial-to-trial adaptations in behavior are related to effort recruitment as measured with pupil dilation. However, it is not yet clear when this adaptation occurs. Whereas the original computational implementation of conflict monitoring theory suggests that conflict from a previous trial starts to recruit effort in the subsequent trial (Botvinick et al., 2001), other models suggest that the adaptation of control may already start to develop within the previous conflict trial itself (Brown et al., 2007; Goschke and Dreisbach, 2008; Scherbaum et al., 2011, 2012). The pupil dilation data currently available are compatible with both accounts (cf. van Steenbergen and Band, 2013).

In order to test the effects of task difficulty on sequence effects in cognitive control and effort mobilization, we conducted two new experiments that included a flanker and a Stroop task where pupil data was also acquired. Experiments 2 and 3 included a flanker and a Stroop task similar to those used in Experiment 1. In Experiment 2, we expected to replicate the behavioral observation of Experiment 1. That is, in comparison to the flanker task, the Stroop task (reported to be more difficult in Experiment 1) is expected to produce smaller or absent conflict-adaptation effects. Pupil dilation data were analyzed to explore whether differences in behavioral adaptation are associated with different effort mobilization as a function of current and previous trial conflict.

### Methods

#### Participants

Twenty-eight healthy right-handed Dutch students participated either for payment or course credits (18–30 years old; seven males). All participants indicated not to use medication (other than anti-conception pills) and were not color blind. Four participants were excluded from analysis because of technical problems during the data acquisition. After initial data screening, two other participants were excluded because of random performance in one or more of the task blocks. The experiment was conducted in accordance with relevant laws and institutional guidelines and was approved by the local ethics committee from the Faculty of Social Sciences.

#### Tasks

The flanker and Stroop tasks were identical to those used in the pilot study with a few exceptions. First, the Stroop task only included one stimulus rather than a column of five identical stimuli in order to prevent potential dilution-effect confounds (cf. Kahneman and Chajczyk, 1983). Second, because we aimed to match the luminance of the ink colors for the Stroop tasks, we selected colors from the isoluminant set of Teufel colors (Teufel and Wehrhahn, 2000). Because there were not two sets of four unique colors from the Teufel colors available that were approximately the same in the number of characters and frequency of the corresponding color word, we decided to use a fixed set of color words for the flanker and Stroop task. To specify, the flanker task always used the words “brown,” “gray,” “yellow,” and “red” whereas the Stroop tasks always used the words “purple,” “green,” “orange,” and “blue” (all words were presented in Dutch translations). Isoluminant ink colors from the Teufel colors set were used for the Stroop task (Teufel and Wehrhahn, 2000) whereas the flanker task stimuli were printed in black. Finally, in order to avoid pupil light reflexes produced by stimulus presentation (cf. Beatty and Lucero-Wagoner, 2000) a scrambled picture of the average stimulus was used as a baseline fixation stimulus (for both tasks separately).

#### Procedure

After informed consent was given, participants were seated in a dimly lit room where the eye tracker was calibrated. Following a data quality check, participants performed 28 practice trials for both tasks which were repeated until they sufficiently learned the task to start the experiment proper. Flanker and Stroop trials were presented in 12 alternating blocks (in counterbalanced order). Before each block started, a self-paced textual reminder of the stimulus-response mapping was shown for a maximum of 15 s. Each block consisted of 36 consecutive test trials (see under Tasks) and 18 consecutive filler trials with a constant inter-trial interval of 4 s (test and filler sequence in random order). For both the flanker and Stroop task, 216 test trials were available for sequential analyses of RT and pupil dilation. The 108 filler trials were used to validate the timing of the pupil dilation response in the test trials.

Following each block, participants received accuracy feedback about their performance in a line graph showing their accuracy per block over time. Feedback was given for the flanker and Stroop task separately. Participants were required to make errors within a target range of 5–10%, and if the participant reached this target they received positive feedback which still encouraged both speed and accuracy. If the error rate dropped below 5%, participants received the following text feedback: “You are not doing your best. Please increase speed. You are allowed to make more errors.” If the error rate exceeded 10%, participants received the following text feedback: “You are not doing your best. You are making too many errors. Please improve accuracy but keep responding fast.” A reminder of the feedback given earlier was provided again at the start of the next task block. Visual feedback was verbally reinforced by the experimenter. Short self-paced breaks (for a maximum of 30 s) were provided following each pair of two blocks. Participants had a fixed 1-min break halfway the experiment.

At the end of the experiment, participants evaluated the flanker and Stroop task in terms of weariness, unpleasantness and difficulty on a 6-points scale. However, these ratings were not assessed separately for the filler trials and test trials, which might potentially limit their usefulness.

#### Pupil Data Acquisition and Analysis

Pupil diameter was recorded at 60 Hz using a Tobii T120 eye tracker, which is integrated into a 17-inch TFT monitor. Participants were seated at a distance of ∼60 cm from the monitor. Pupil data were processed and analyzed using custom-made macros programmed in Brain Vision Analyzer. The artifacts and eye blinks that were detected by the Tobii eye tracker plus three samples before and after these data points were marked as missing data. These samples were corrected using linear interpolation. In order to reduce the impact of potential outliers due to unreliable pupil data interpolation, while at the same time keeping sufficient trials to reach a good signal-to-noise ratio, trials with less than 20% data points obtained in the intervals of interest were excluded from analyses. After visual inspection (see below), pupil dilation was defined as the mean pupil diameter during a 700–1300 ms period following stimulus onset minus the mean pupil diameter in the baseline interval. A 200-ms pre-stimulus interval was used as baseline.

### Results

Behavioral and pupil analyses reported for Experiments 2 and 3 were performed after the following trials were excluded: the first trial of each block, trials following an error, trials with RTs not fitting the outlier criterion (2.5 SDs deviating from the individual condition-specific mean), and trials including unreliable pupil-data interpolations.

#### Subjective Reports

Unlike the findings in Experiment 1, rated task difficulty for the Stroop task was not significantly higher in comparison to the flanker task [4.6 versus 4.1; *t*(21) = 1.1, *p* = 0.282]. Weariness and unpleasantness scores were also not different for the tasks [*t*(21)s < 1.3, *p*s > 0.23].

#### Behavioral Results

Correct RT data are shown in **Figure 1**. We first ran analyses on the two tasks separately. Replicating our pilot study, both the flanker and the Stroop task yielded a congruency effect [24 ms; *F*(1,21) = 67.7, *p* < 0.001, MSE = 184.9 and 35 ms; *F*(1,21) = 17.1, *p* < 0.001, MSE = 1538.0], which was modulated by previous trial conflict in the flanker task [19 ms; *F*(1,21) = 9.7, *p* < 0.01, MSE = 213.6], but not in the Stroop task [6 ms; *F*(1,21) = 0.4, *p* = 0.52, MSE = 456.0]. Error rate data revealed congruency-effects for the flanker [2.7%; *F*(1,21) = 6.1, *p* < 0.03, MSE = 0.003] and the Stroop task [1.5%; *F*(1,21) = 4.4, *p* < 0.05, MSE = 0.001] but no indications of conflict adaptation for both task (-1.6 versus 1.1%; *F*s < 1). These behavioral results replicate the finding in Experiment 1: the Stroop task produced smaller congruency-sequence effects than the flanker task.

An additional ANOVA was run to statistically compare the effects of task directly. This analysis revealed a main effect of task on RT, *F*(1,21) = 62.7, *p* < 0.001, MSE = 1358.4, and accuracy, *F*(1,21) = 4.4, *p* = 0.049, MSE = 0.01, indicating faster responses (509 versus 553 ms) and less errors (6.3 versus 7.5%) on the Stroop task than on the flanker task. There was no evidence that task interacted with the congruency-sequence effect in RT, *F*(1,21) = 1.4, *p* = 0.253, MSE = 359.3, or accuracy, *F*(1,21) = 1.2, *p* = 0.277, MSE = 0.01.

#### Pupil Data Validation

**FIGURE 2:**
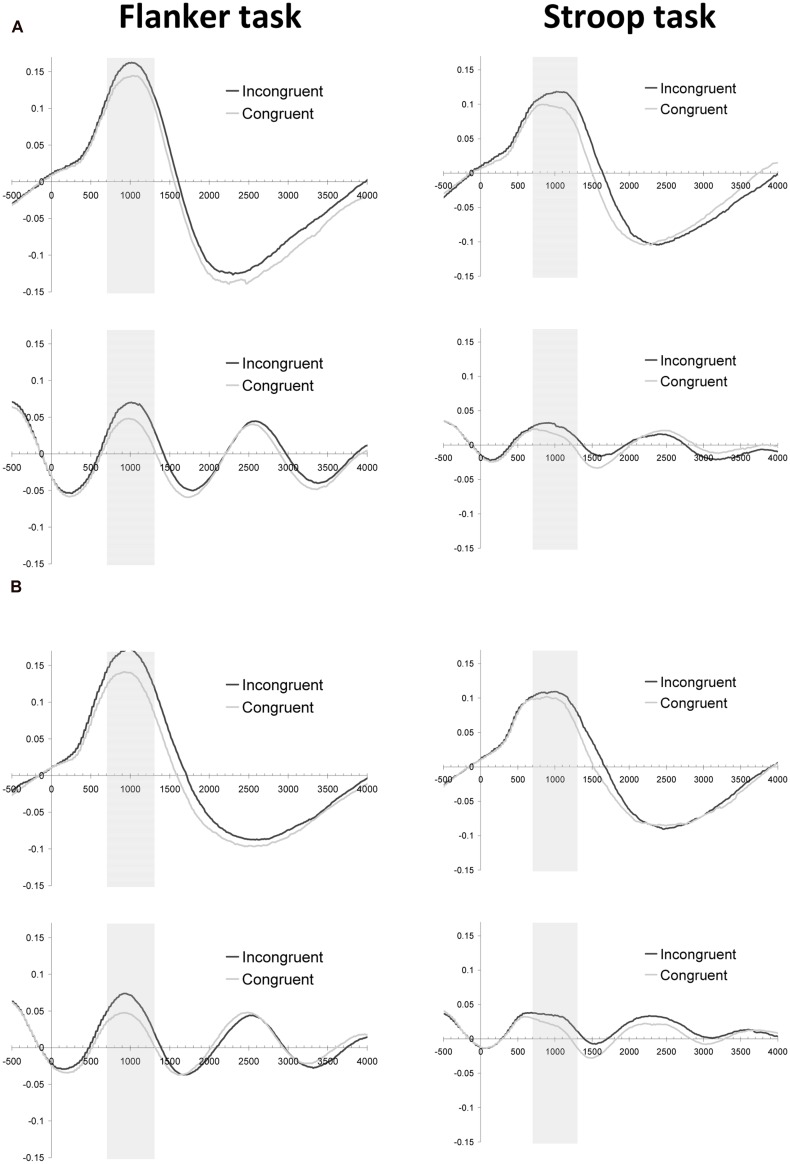
**Pupillary response (mm) as a function of time (ms) and current-trial congruency for Experiment 2 **(A)** and Experiment 3 (B).** Both graphs depict baseline-corrected pupil dilation in the Flanker (left column) and Stroop (right column) task for filler trials (upper row) and test trials (lower row).

In order to explore whether effort mobilization as measured by pupil dilation is different between the flanker and the Stroop task, we measured pupil dilation in response to stimulus onset. As is shown in **Figure 2A** (upper panels), the long-interval filler trials showed a pupil dilation for both the flanker and the Stroop task, which reached its peak value around 1 s after stimulus onset. More importantly, dilations in the same time interval were found for the test trials (the trials with the short inter-trial intervals), validating our approach to measure peak pupil dilation in the 700–1300 ms period^1^ (**Figure 2A**, lower panels).

The pupil dilation data yielded congruency effects in dilation for both the flanker task [*F*(1,21) = 14.5, *p* < 0.001, MSE = 0.001] and the Stroop task [*F*(1,21) = 4.3, *p* = 0.052, MSE = 0.001], irrespectively of the inter-trial interval used (*F*s < 1). Thus, pupil diameter could reliably be used as an index of effort mobilization during the test trials, even though a short inter-trial interval was used (for a similar finding, see van Steenbergen and Band, 2013).

#### Pupil Results

**FIGURE 3:**
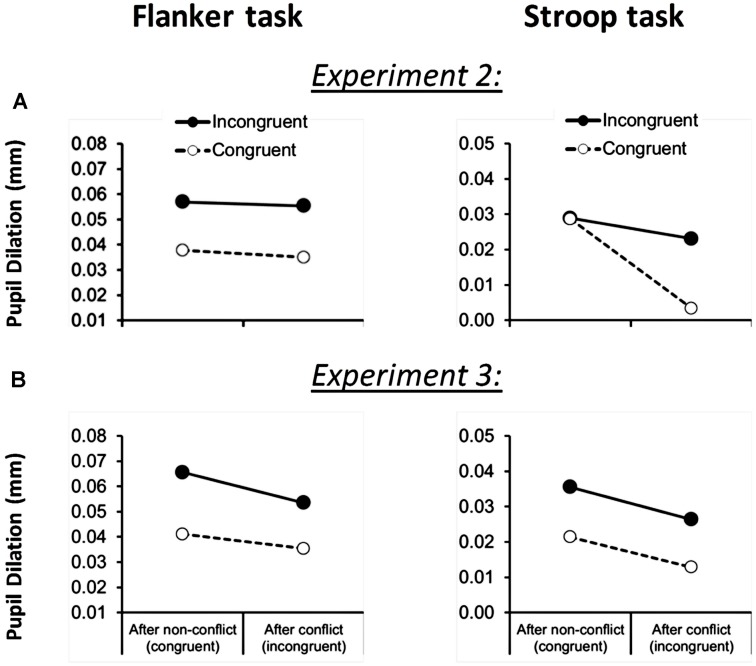
**Flanker task (left column) and Stroop task (right column) pupil dilation across Experiment 2 **(A)** and 3 **(B)**, as a function of current-trial congruency and previous-trial congruency.** Please note that data from the Stroop task in Experiment 2 suggests that there is a current-trial congruency × previous-trial congruency interaction effect. However the task × current-trial congruency × previous-trial congruency interaction effect is not significant.

In order to explore differential effort mobilization in the Stroop versus the flanker task, we analyzed pupil dilation during test trials as a function of congruency of the current trial and congruency of the previous trial, using task (flanker versus Stroop) as an additional within-subject factor. As shown in **Figure 3A**, both tasks showed more dilation during incongruent trials in comparison to congruent trials [0.015 mm; *F*(1,21) = 10.1, *p* < 0.005, MSE = 0.001]. Independent of this, a trend for a main effect of previous-trial congruency was observed: decreases in current-trial dilations were observed when the previous trial was incongruent [*F*(1,21) = 3.4, *p* = 0.08, MSE = 0.001]. This effect was moderated by a significant Task × Previous-Trial Congruency interaction [*F*(1,21) = 4.7, *p* < 0.05, MSE = 0.0004] showing that the decrease in overall dilation following conflict was only significant in the Stroop task [0.016 mm; *F*(1,21) = 4.9, *p* < 0.05, MSE = 0.001] but not in the flanker task [0.002 mm; *F*(1,21) = 0.30, *p* = 0.60, MSE = 0.0004]. There was no task × current-trial congruency × previous-trial congruency interaction, *F*(1,21) = 1.4, *p* = 0.253, MSE = 359.3. Task also did not significantly interact with other (combinations of) factors.

Because the measure of pupil diameter in the current trial was corrected for the pre-stimulus baseline in that trial, the earlier analyses on pupil dilation were not sensitive to after-effects of pupil dilation starting in the previous trial. We therefore also analyzed raw pupil diameter in the baseline interval of the current trial. The Task × Previous-trial Congruency effect observed in pupil dilation was not found in this baseline interval [*F*(1,21) = .55, *p* = 0.47, MSE = 0.002].

#### Relationship between Perceived Task Difficulty and Conflict Adaptation

**FIGURE 4:**
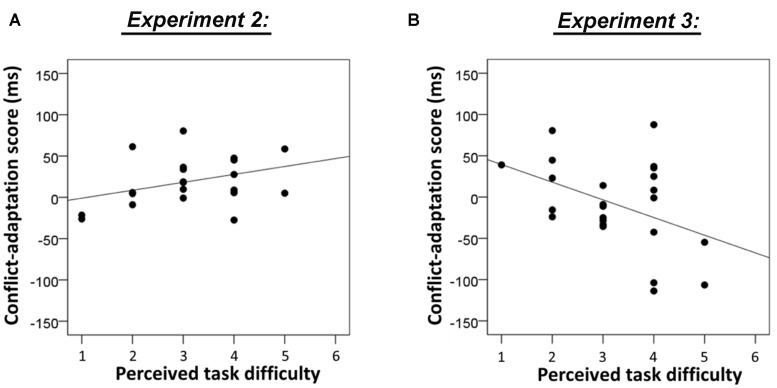
**(A)** Association between individual task difficulty ratings and conflict-adaptation scores of the flanker task in Experiment 2. Note that this correlation is not significant (*p* = 0.091). **(B)** Association between individual task difficulty ratings and conflict-adaptation scores of the flanker task in Experiment 3 (*p* = 0.043).

In order to investigate whether individual differences in perceived task difficulty predicted the size of the conflict adaptation effects in this sample, we again regressed individual task difficulty ratings on individual scores of conflict adaptation separately for the flanker task and the Stroop task. Given the hypothesized inverted-U relationship between task difficulty and conflict adaptation, a positive correlation might be observed for the easiest task. Indeed, there was a trend for a positive linear relationship between task difficulty and conflict adaptation on the flanker task, *R*^2^ = 0.136, *F*(1,20) = 3.2, *p* = 0.091, see **Figure 4A**. For the Stroop task there was no evidence for an association (*F*s < 1).

### Discussion

Experiment 2 replicated the behavioral effect in Experiment 1: conflict adaptation was reduced in the task reported to be more difficult in Experiment 1. Pupil data across the flanker and Stroop task showed that incongruent trials produced more dilation than congruent trials. Thus, replicating and extending earlier studies, both Stroop conflict and flanker conflict induced pupil dilation. More importantly, sequential analyses of pupil dilation revealed a main effect of previous trial congruency in the more difficult task only. That is, previous-trial conflict reduced pupil dilation in the subsequent trial only in the task that did not show conflict adaptation (Stroop task), whereas there was no evidence for a previous-trial effect in the task that did produce conflict adaptation (flanker task). The latter finding is consistent with an earlier study that also showed behavioral conflict adaptation in the absence of a main effect of previous-trial conflict in a Simon task (van Steenbergen and Band, 2013). It could be speculated that the reduced post-conflict pupil dilation observed in the context of an absent behavioral congruency-sequence effect reflects a physiological marker of mental overload. This working hypothesis is consistent with the demand-driven decline of pupil dilation reported earlier for mental-overload conditions in other paradigms, including a RT task using extreme presentation rates (Poock, 1973) and a digital span recall task using excessive load (Granholm et al., 1996).

Although subjective reports from Experiment 1 support our claim that the Stroop task was perceived to be more difficult than the flanker task, we failed to observe differences in perceived difficulty in Experiment 2. In addition, the correlational analysis only showed a trend for a positive relationship between task difficulty and conflict adaptation in the flanker task. These null-finding might be attributed to the sample size employed in Experiment 2, which was much smaller than in Experiment 1. However, we admit that it is also well possible that other factors might have produced the differences in behavioral conflict adaptation. Although we carefully matched the Stroop and flanker tasks in terms of stimulus material (i.e., color words) and the manual response required, earlier work indeed have suggested that different conflict paradigm might involve independent, domain-specific cognitive and neural mechanisms (Egner, 2008). Although both the Stroop and the flanker task are thought to induce conflict between relevant and irrelevant stimulus dimensions (Kornblum et al., 1990; Egner, 2008), and pupillary responses and behavior confirmed comparable congruency effects, the source of this conflict likely is different. For example, according to the Dimension-Action model (Magen and Cohen, 2002) the Stroop effect is driven by conflict between relevant and irrelevant verbal codes (following the translation from color to word), whereas conflict in the flanker task takes place between relevant and irrelevant stimulus elements in a visual dimension. In addition, Stroop interference stems from two features of the same visual object, whereas flanker interference stems from features of different visual objects (Magen and Cohen, 2002). This may have differentially impacted perceived task demands. Processing the relevant word color in a Stroop stimulus is inevitably accompanied by the processing of the irrelevant color word (Duncan, 1984; Chen, 2003), which may induce task conflict even in congruent trials (Goldfarb and Henik, 2007). In contrast, selective processing of central target relative to surrounding stimuli in the flanker task is simply possible through a spatial narrowing of attention (Laberge et al., 1991). One or more of these factors might have produced an increase in the reported level of task difficulty for the Stroop task.

As a side note, we want to emphasize that a common interpretation of slower RT and/or increased congruency effects in terms of increased difficulty is not consistent with the observation in the previous experiments. Instead, the task rated to be more difficult (in Experiment 1) was associated with faster responses. However, note that some studies in fact have observed faster responses as a consequence of the participant’s adaptation to higher levels of task difficulty (cf. Washburn and Putney, 2001). Since we compared two different tasks requiring different processing of relevant and irrelevant perceptual features, it is obvious that faster RTs can also be attributed to more efficient processing in processing stages prior to response selection, such as perceptual processes. Moreover, because we used fixed response–stimulus intervals, faster RTs may have increased stimulus pacing, which in turn could have increased task difficulty.

It is also important to stress that conflict-adaptation effects *have* been demonstrated in numerous studies on Stroop and Stroop-like paradigms (Egner and Hirsch, 2005b; Naccache et al., 2005; Verbruggen et al., 2006; Duthoo and Notebaert, 2012; Puccioni and Vallesi, 2012; Blais et al., 2014), although it is not clear how demanding these tasks actually are. In addition, some previous studies used simple categorization responses and/or a low ratio of incongruent trials (e.g., Kerns et al., 2004), which may well have inflated adaptation effects (cf. Purmann et al., 2009).

In sum, it is clear that Experiments 1 and 2 only provide correlational evidence for a link between task difficulty and conflict-adaptation reductions. Since we used different paradigms (Stroop versus flanker) it is difficult to exclude alternative explanations in terms of domain-specific mechanisms. In addition, although in both Experiments 1 and 2 the flanker conflict adaptation was significant for the flanker task, but not for the Stroop task, the actual difference in conflict-adaptation size between tasks was not significant, which prevents drawing strong inferences from the pattern of data observed (for an instructive overview of this issue, see Nieuwenhuis et al., 2011). In Experiment 3 we therefore introduced an experimental manipulation of task demand in order to show that increased task difficulty is associated with reduced conflict adaptation independent of the specific paradigm used. In addition, if the effect of this manipulation is orthogonal to the effect of task type, we should observe a statistically significant reduction of conflict adaptation when comparing Experiment 3 with Experiment 2.

## Experiment 3

In order to increase overall effort mobilization, task demands in Experiment 3 were further increased using a time-pressure manipulation (Kahneman, 1973). A new group of participants performed exactly the same tasks as used in Experiment 2 but with different instructions. Specifically, the instructions emphasized that participants should try improving their speed over the course of the experiment. In order to prevent a shift in speed-accuracy tradeoff participants were encouraged to maintain good accuracy levels. We hypothesized that this additional increase in task difficulty should further reduce congruency-sequence effects in both tasks. That is, in comparison to Experiment 2, we expected to find an additive reduction of conflict adaptation irrespective of the particular task. We also recorded pupil diameter in order to obtain additional evidence for our working hypothesis that in situations of sustained task difficulty, previous-trial conflict reduces pupil dilation in the subsequent trial. Thus, we predicted to observe post-conflict reduction in pupil dilation in the context of tasks where behavioral conflict adaptation is absent.

### Methods

#### Participants

Twenty-seven healthy right-handed Dutch students participated either for payment or course credits (18–30 years old; eight males). All participants indicated not to use medication (other than anti-conception pills) and were not color blind. After initial data screening, three participants were excluded because of random performance in one or more of the task blocks. The experiment was conducted in accordance with relevant laws and institutional guidelines and was approved by the local ethics committee from the Faculty of Social Sciences.

#### Tasks

See Experiment 2.

#### Procedure

Procedures were identical to Experiment 2, except for the performance feedback and instructions that participants received. Like in Experiment 2, after each block, participants received both accuracy and correct RT feedback about their performance in a line graph showing their accuracy and speed per block over time. Feedback was given for the flanker and Stroop task separately. In order to prevent a shift in speed-accuracy tradeoff, participants were required to continuously improve speed over time, while keeping errors within a target range of 5–10%. If the participant attained the accuracy target they received positive feedback, which still instructed to further increase speed without reducing accuracy. If the error rate dropped below 5%, participants received the following text feedback: “You are not doing your best. Please increase speed. You are allowed to make more errors.” If the error rate exceeded 10%, participants received the following text feedback: “You are not doing your best (or you respond TOO fast). You are making too many errors. Please try as hard as you can and improve accuracy.” A reminder of the feedback given earlier was provided again at the start of the next task block. Visual feedback was verbally reinforced by the experimenter.

#### Pupil data acquisition and analysis

See Experiment 1.

### Results

#### Subjective Reports

Rated task difficulty for the Stroop task was not significantly different from the flanker task [4.2 versus 4.3; *t*(23) = 0.15, *p* = 0.880]. Weariness and unpleasantness scores were also not different for the tasks [*t*(23)s < 1.7, *p*s > 0.10].

In order to directly compare reported task difficulty in Experiment 3 to Experiment 2, we run an additional ANOVA with experiment as a between-subject factor. This analyses did not provide evidence that task difficulty was perceived higher for Experiment 3, *F*(1,44) = 0.2, *p* = 0.632, MSE = 1.2. There was also no main effect of task, *F*(1,44) = 0.7, *p* = 0.423, MSE = 1.2, or an interaction between experiment and task, *F*(1,44) = 1.0, *p* = 0.327, MSE = 1.2.

#### Behavioral Results

Correct RT data are shown in **Figure 1**. We first ran analyses on the two tasks separately. Consistent with predictions, reduced conflict adaptation was observed across tasks. No evidence for a congruency-sequence effect was found for the flanker task [-9 ms; *F*(1,23) = 0.66, *p* = 0.43, MSE = 701.6], whereas the Stroop task showed a reversal of the congruency-sequence effect [-15 ms; *F*(1,23) = 7.7, *p* < 0.02, MSE = 187.1]. Congruency effects [35 ms, *F*(1,23) = 27.1, *p* < 0.001, MSE = 385.8 and 21 ms, *F*(1,23) = 13.9, *p* < 0.001, MSE = 2166.2] were similar to those reported for Experiment 2. As in Experiment 2, error rate data revealed significant flanker and Stroop congruency-effects [0.5%, *F*(1,23) = 6.5, *p* < 0.02, MSE = 0.002 and 2.7%, *F*(1,23) = 9.6, *p* < 0.005] but no indications of conflict adaptation for both task (0.6 and 0.9%, *F*s < 2). In addition, the Stroop task produced a previous-trial congruency effect on accuracy [*F*(1,23) = 4.6, *p* < 0.05, MSE = 0.001], showing an increase in error rate (1.4%) after conflict in the previous trial.

An additional ANOVA was run to statically compare the effects of task directly. This analysis revealed a main effect of task on RT, *F*(1,23) = 12.4, *p* = 0.002, MSE = 6052.9, and accuracy, *F*(1,23) = 8.5, *p* = 0.008, MSE = 0.01, indicating faster responses (507 versus 546 ms) and less errors (6.5 versus 8.4%) on the Stroop task than on the flanker task. There was no evidence that task interacted with the congruency-sequence effect in RT, *F*(1,21) = 1.4, *p* = 0.253, MSE = 359.3, or accuracy, *F*(1,21) = 1.2, *p* = 0.277, MSE = 0.01. The interaction between task and previous-trial congruency effect on accuracy was also significant, *F*(1,23) = 5.2, *p* = 0.032, MSE = 0.01.

In order to directly compare the behavioral findings of Experiment 3 to Experiment 2, we run an ANOVA with experiment as a between-subject factor. The only significant effects observed between experiment and (combinations of) other factors was an interaction between experiment and the congruency-sequence effect, *F*(1,44) = 7.9, *p* = 0.007, MSE = 451.6, confirming that the manipulation in Experiment 3 successfully reduced conflict adaptation (orthogonal to task). There was no evidence for a main effect of experiment on RT or accuracy, indicating that no shift in speed-accuracy trade-off was induced by our manipulation (*F*s < 1).

#### Pupil Data Validation

As **Figure 2B** shows, the dilation patterns in Experiment 3 mirrored the effects observed in Experiment 2. Congruency effects were observed in the flanker task [*F*(1,23) = 26.5, *p* < 0.001, MSE = 0.001] and in the Stroop task [*F*(1,23) = 4.0, *p* = 0.059, MSE = 0.002], irrespectively of the inter-trial interval used (*F*s < 1).

#### Pupil Results

As shown in **Figure 3B**, both tasks caused more dilation to incongruent than to congruent test trials [0.017 mm; *F*(1,23) = 16.3, *p* < 0.001, MSE = 0.001]. Independent of this, previous-trial congruency also influenced current-trial dilations: decreases in dilation were observed when the previous trial was incongruent [0.009 mm for both tasks; *F*(1,23) = 8.8, *p* < 0.01, MSE = 0.0004], irrespectively of task type (*F* < 1). There was no task × current-trial congruency x previous-trail congruency interaction, *F*(1,23) = 0.3, *p* = 0.619, MSE = 0.01.

We also directly compared pupil dilation results in Experiment 3 to Experiment 2, running an additional ANOVA with experiment as a between-subject factor. This analyses confirmed the previous congruency effect, *F*(1,44) = 10.1, *p* = 0.003, MSE = 0.001, and also revealed main effects of task, *F*(1,44) = 32.2, *p* < 0.001, MSE = 0.002, and congruency, *F*(1,44) = 25.8, *p* < 0.001, MSE = 0.001. However, the interaction between previous congruency and experiment was not significant, *F*(1,44) = 1.6, *p* = 0.208, MSE = 0.001.

#### Relationship between Perceived Task Difficulty and Conflict Adaptation

In order to test whether the assumed increase in difficulty in this Experiment produced a shift to the right side of the inverted U-shape between difficulty and conflict adaptation, we again regressed individual task difficulty ratings on individual scores of conflict adaptation separately for the flanker task and the Stroop task. Interestingly, there was a significant *negative* linear relationship between task difficulty and conflict adaptation on the flanker task, *R*^2^ = 0.174, *F*(1,22) = 4.6, *p* = 0.043, see **Figure 4B**, but no evidence for an effect on the Stroop task (*F*s < 1).

### Discussion

As expected, conflict-adaptation effects in Experiment 3 were eliminated for both tasks when performed under conditions of increased task difficulty using time pressure. The analysis that directly compared Experiment 3 to Experiment 2 revealed that our manipulation led to an overall reduction of conflict adaptation, whereas it did not affect other aspects of behavior, such as overall speed and accuracy and the congruency effects thereof. This suggests that our time-pressure manipulation indeed successfully prevented a shift in speed-accuracy tradeoff and selectively modulated conflict adaptation only. Indeed, standard congruency effects in RT and pupil dilation in Experiment 3 suggested that these tasks still induced response conflict and effort mobilization.

However, when comparing task difficulty ratings of Experiment 3 to Experiment 2 directly, no significant difference in perceived difficulty was observed. It is difficult to unequivocally interpret this null-finding. On the one hand, reported task difficulty might have been less sensitive to small differences because it was assessed at the task level only. Moreover, our comparison involved different groups of participants who may have interpreted the scale relatively to the difference between the flanker and Stroop tasks they just performed (i.e., anchoring; Tversky and Kahneman, 1974) instead of providing an objective context-independent measure of perceived task difficulty. On the other hand, our data also leaves the possibility open that time pressure actually did not change perceived task difficulty. Instead, time pressure might have lowered the expectancy of success, thus lowering the level of potential motivation. According to motivation intensity theory (Brehm and Self, 1989) and related motivational accounts (Kahneman, 1973; Tops et al., 2015), reduced potential motivation lowers the upper limit of the curvilinear relationship between perceived difficulty and effort, which can reverse the relationship between task difficulty and effort.

**FIGURE 5:**
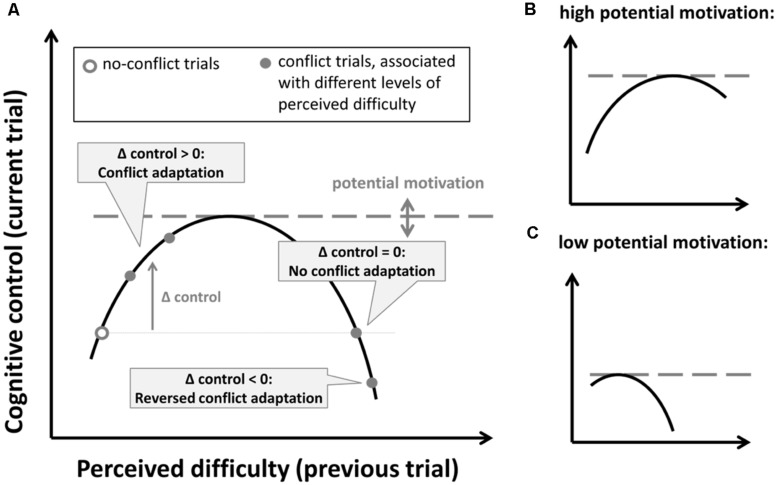
**(A)** Hypothesized inverted U-shape relationship between perceived difficulty and subsequent increases in cognitive control. The level of potential motivation determines the upper limit of the curve. **(B,C)** Illustration of this relationship for a high **(B)** and low **(C)** level of potential motivation.

The proposed inverted-U relationship between task difficulty and cognitive control that follows from these motivational accounts is depicted in **Figure 5A**. When overall task difficulty is high, it is likely that conflict trials are perceived to be more difficult, which in turn increases or decreases conflict-adaptation, depending on the side of curve. If this is true, the reduced conflict adaptation in the more difficult tasks is produced by an increase in task difficulty beyond the apex of the curve (see **Figure 5**). A further increase in task difficulty might account for the small reversal of conflict adaptation of Stroop performance observed under time pressure in Experiment 3. Alternatively, in the absence of significant differences in self-reported task difficulty, the same effects can also be accounted for by a shift in the inverted U-shape due to a reduction of potential motivation. This is illustrated in **Figures 5B,C**. Note that the positive versus negative relationship observed under respectively the normal versus time-pressure conditions (see **Figure 4**) also fits this account.

The pupil dilation data from Experiment 3 was consistent with the observation for the Stroop task in Experiment 2: The absence of behavioral adaptation in both tasks was accompanied by a conflict-driven reduction in pupil dilation in both tasks. However, when directly comparing the pupil dilation effects in Experiments 2 and 3, we did not find evidence for the idea that conflict-driven dilation reduction was further increased in Experiment 3. Thus, the effect size of previous-trial conflict on pupil dilation did not scale with the reduction in conflict adaptation observed. Although it is too early to draw strong conclusions, these findings hint at the possibility that dilation reduction might be useful as a binary marker of whether or not mental overload occurs, but that it does not reflect the size of this overload (i.e., the distance between available and required resources).

However, weaknesses of the previous experiments reported so far seriously limit the inferences that can be made from this set of observations. For example, a statistical difference in conflict adaptation was only observed when comparing Experiments 2 and 3 directly, whereas the difference in conflict adaptation between the flanker and Stroop task was only at trend level in Experiment 1 and not significant in Experiments 2 and 3. So, our design might have been underpowered and it is possible that similar Stroop tasks reliably induce conflict-adaptation effects when bigger samples are used. A more important limitation is that task difficulty was assessed at the task level only and the observed effects on task difficulty were small (Experiment 1) or absent (Experiments 2 and 3). Moreover, the associations between rated task difficulty and conflict adaptation were not strong: we failed to observe a relationship for the Stroop task, whereas there was limited evidence for an association in the flanker task (a trend in Experiment 2 and a small significant effect in Experiment 3).

## Experiment 4

Given these considerations, we run a new behavioral study that utilized an improved assessment of perceived difficulty in a large sample of participants that was run online. To specify, after participants performed the flanker and Stroop task, they were asked to rate the perceived difficulty of each of the trial types of both tasks. This self-report data allowed us to run analyses that focused on the rated difficulty of conflict and no-conflict trials, rather than performing analysis on the level of overall task difficulty. We expected that this more fine-grained analysis combined with a large sample (*N* = 155) would help to (1) provide additional evidence for the hypothesis that Stroop conflict is perceived as more difficult than flanker conflict, and (2) to provide converging evidence for an inverted U-shape relationship between difficulty at the level of no-conflict and conflict trials.

### Methods

#### Participants

Participants were recruited through Amazon Mechanical Turk (AMT) and were required to be located in the USA. One hundred and ninety eight participants completed the task [19–69 years old (*M* = 36), 62 males]. An initial screening survey ensured that participants could only participate if they were not color blind. Thirteen participants were excluded from analysis because of technical problems during the data acquisition (Qualtrics server was temporarily not available during a block of trials). After initial screening of behavioral data, eight and fifteen other participants were excluded because of more than 20% response omissions and random performance in one or more of the task blocks, respectively. Finally, datasets from seven other participants were excluded because the recorded inter-trail interval exceeded the intended duration with more than 2 s for more than 25% of the time in one or more of the task blocks. The experiment was conducted in accordance with relevant laws and institutional guidelines and was approved by the local ethics committee from the Faculty of Social Sciences.

#### Tasks

The flanker and Stroop tasks were identical to those used in the Experiment 1 with a few exceptions. First, given that the participants were located in the USA, English instead of Dutch color words were presented. Second, for this online study we used the QRTEngine (version 16, preloading next trial enabled) for stimulus presentation and response recording (Barnhoorn et al., 2015). Stimuli appeared in lower-case in 38-pt Arial bold font and were presented on a white background.

#### Procedure

After testing the estimated server communication delay, and ensuring that it was lower than 2000 ms (Barnhoorn et al., 2015), participant were informed about the task, gave informed consent and did a brief test for color blindness. They then got detailed instructions about the flanker and Stroop tasks and participants performed 12 practice trials with error feedback for both tasks. Participants then performed two alternating runs of a flanker and a Stroop task block (in counterbalanced order). A textual reminder of the stimulus-response mapping was shown before the start of each of the four blocks of 72 trials. At the end of the experiment, we presented all 12 stimuli associated with the different trial types, separately for the flanker and Stroop task, and asked participants to rate how difficult – on average – it appeared to them to respond quickly and accurately to the respective trial when that stimulus was presented in the two task blocks of the task they did before. Participants reported perceived difficulty on a 9-points scale ranging from very easy to very difficult.

### Results

#### Subjective Reports

Analyses were run on the mean difficulty rating of all conflict (incongruent) and no-conflict (congruent) trials, for the Stroop and flanker task separately. In line with the task difficulty ratings of Experiments 1 and 2, a main effect of task revealed that trials in the Stroop task were rated to be more difficult than the flanker task [3.6 versus 3.2; *F*(1,154 = 10.2, *p* = 0.002, MSE = 2.3]. A main effect of congruency showed that incongruent trials were rated to be more difficult than congruent trials [4.2 versus 2.6; *F*(1,154 = 260.7, *p* < 0.001, MSE = 1.7]. Critically, there was also an interaction between task and congruency [*F*(1,154 = 12.7, *p* < 0.001, MSE = 0.7], showing that incongruent Stroop trials were reported to be more difficult than incongruent flanker trials [4.5 versus 3.9; *t*(154) = 4.0, *p* < 0.001] whereas congruent Stroop and flanker trials did not differ significantly on perceived difficulty [2.6 versus 2.5; *t*(154) = 1.2, *p* = 0.247].

#### Behavioral Results

The first trial of each block and trials not complying with the outlier criterion (2.5 SDs) were excluded from all analyses. ANOVAs on correct RT data revealed significant basic congruency effects for both the flanker task [28 ms; *F*(1,154) = 144.8, *p* < 0.001, MSE = 809.9] and the Stroop task [55 ms; *F*(1,154) = 175.8, *p* < 0.001, MSE = 2652.2]. Unlike in the previous experiments, however, both tasks produced congruency-sequence effects (see **Figure 1**). That is, a significant reduction of the congruency effect following conflict was found for the Stroop task [14 ms; *F*(1,154) = 7.1, *p* = 0.009, MSE = 1021.0] and a tendency for this effect was observed in the flanker task [9 ms; *F*(1,154) = 3.5, *p* = 0.064, MSE = 806.0]. Accuracy data also showed basic congruency effects for the Stroop task [2.7%; *F*(1,154) = 33.1, *p* < 0.001, MSE = 0.01] and a trend for the flanker task [0.5%; *F*(1,154) = 2.8, *p* = 0.094, MSE = 0.01]. The congruency-sequence effect in accuracy was not significant for both tasks (*F*s < 2.7).

An additional ANOVA was run to statically compare the effects of task directly. Task did not interact with the congruency-sequence effect in RT or accuracy (*F*s < 1). Task did interact with the congruency effect in RT [*F*(1,154) = 31.2, *p* < 0.001, MSE = 1858.5] and accuracy [*F*(1,154) = 16.2, *p* < 0.001, MSE = 0.01], indicating that the congruency effects were significantly larger in the Stroop task than in the flanker task.

#### Relationship between Perceived Task Difficulty and Conflict Adaptation

In order to investigate whether individual differences in the size of the difference between perceived difficulty of conflict and no-conflict trials predicted the size of the conflict adaptation effects, we regressed the difference between the mean rated difficulty of incongruent minus congruent trials on individual scores of conflict adaptation, separately for the flanker task and the Stroop task, using linear and quadratic curve fitting as implemented in SPSS. Initial screening of the scatter plots revealed one outlying participant that rated the incongruent flanker stimuli as 3 points easier than congruent flanker stimuli. This participant was excluded from all subsequent analyses.

**FIGURE 6:**
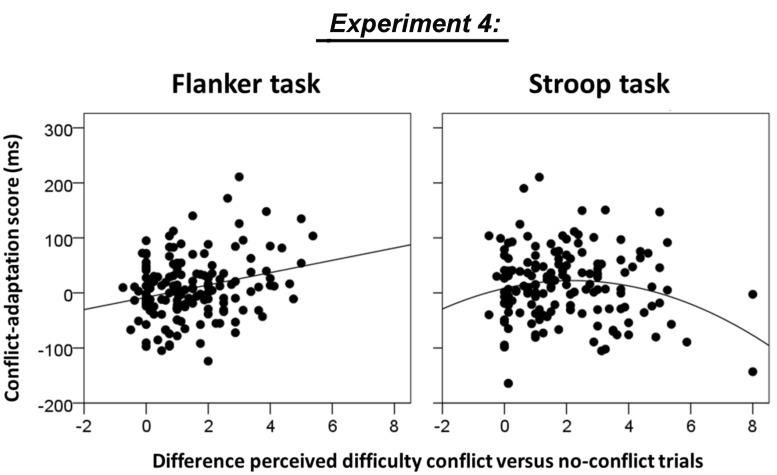
**Association between the difference in rated difficulty of conflict minus no-conflict trials and conflict adaptation in Experiment 4 for the flanker task (linear model fit, *p* = 0.001) and the Stroop task (quadratic model fit, *p* = 0.049).** Please note that the quadratic fit for the Stroop task did not remain significant after removing the two potential outliers with high difficulty ratings.

For the flanker task, both the linear model and the quadratic model fitted the data well, *F*(1,152) = 11.3, *p* = 0.001, *R*^2^= 0.069 and [*F*(2,151) = 6.8, *p* = 0.001, *R*^2^ = 0.083]. However, since the coefficient of the quadratic term in the quadratic model was not significantly different from zero (β = 0.319, *p* = 0.138), the model that assumed a linear relationship between perceived task difficulty and conflict adaptation was the most parsimonious (see **Figure 6**, left panel). For the Stroop task, the quadratic model but not the linear model fitted the data, *F*(2,151) = 3.1, *p* = 0.049, *R*^2^ = 0.039 versus [*F*(1,152) = 0.9, *p* = 0.324, *R*^2^ = 0.006, see **Figure 6**, right panel. Only the quadratic term was significantly different from zero (β = -0.458, *p* = 0.024). However, note that the evidence for a quadratic relationship is not strong since the model fit did not remain significant after removing two potential outliers with high difficulty ratings (difference score = 8, see **Figure 6** right panel).

### Discussion

Experiment 4 provided converging evidence for the hypothesis that the Stroop task, more specifically the conflict trials in this task, was perceived to be more difficult than the flanker task when tested online in a large sample of AMT workers. Interestingly, this experiment also revealed a significant conflict adaptation effect in the Stroop task that did not yield a reliable adaptation effect in the Experiments 1, 2, and 3 run in our local behavioral lab. In comparison to these previous studies, increased overall RTs and lower error rates were observed in this online study, even though instructions emphasized that both speed and accuracy were important. This shift in speed-accuracy trade-off might also account for the differences in congruency and adaptation effects observed. These findings mainly underscore that instructions in online experiments may be interpreted differently, likely because there is no experimenter that can supervise task performance.

These limitations notwithstanding, the regression analyses in Experiment 4 clearly showed a positive association between perceived difficulty and conflict adaptation in the flanker task (**Figure 6** left panel), which replicated the trend effect observed in Experiment 2 for the same task (**Figure 4A**). A linear relationship was absent in the Stroop task, presumably because these difficulty scores are distributed around the apex of the U-shape. Indeed, a U-shaped model was shown to fit the scatter although the limited number of extreme difficulty ratings made it difficult to decide whether this analysis revealed a real effect or whether it should be attributed to the presence of potential outliers.

## General Discussion

In a series of four experiments we aimed to show that tasks associated with higher levels of reported task difficulty are associated with decreased conflict adaptation. The results provide limited initial support for this hypothesis. Experiment 1 provided some evidence for the idea that performing a Stroop task is perceived as being more difficult than performing a flanker task, a finding that was also confirmed in Experiment 4. However, this increased difficulty was not unequivocally linked to reduced conflict adaptation. Whereas indices of conflict adaptation in the Stroop tasks were less reliable in Experiments 1–3, a direct statistical comparison to the flanker task revelead only a trend for reduced adaptation in Experiment 1. This effect was not reliable replicated in Experiments 2–4, and the results from the online study in Experiment 4 actually showed that normal conflict adaptation can be observed in the Stroop task. Taking together these findings, we thus did not provide consistent evidence that the Stroop tasks produce smaller conflict adaptation effects than the flanker task.

However, orthogonal to the effect of task type, when compared to the results of Experiment 2, the time-pressure manipulations used in Experiment 3 was shown to reduce conflict adaptation. However, perceived difficulty in the group of participants that performed the tasks under time pressure was not significantly higher, so reduced conflict adaptation could also be accounted for by a lowering of potential motivation induced by time pressure (see **Figures 5B,C**). At the same time, however individual differences in self-reported task difficulty in Experiment 2–4 in the flanker task did systematically relate to the size of the conflict-adaptation effect, thus providing at least initial evidence for the proposed inverted-U shape relationship between perceived task difficulty and conflict adaptation. These effects were not consistently observed in the Stroop task (but see Experiment 4), presumably because the difficulty scores were distributed around the apex of the U-shape.

In addition, we showed that pupillometry might provide an interesting tool to index effort mobilization in cognitive control paradigms. In particular, the data from Experiments 2 and 3 provide initial insight in how and when difficult situations in the context of sequential adaptations cause effort mobilization. Firstly, similar to previous findings (van Steenbergen and Band, 2013), behavioral conflict adaptation was not reflected by a temporary increase in effort in the subsequent trial (**Figure 3A**, left figure), which implies that conflict-driven mobilization of effort starts earlier, presumably within the previous conflict trial itself (Scherbaum et al., 2011; for an extended discussion, see van Steenbergen and Band, 2013). Secondly, during the tentatively high-demanding task conditions in Experiment 2 (**Figure 3A**, right figure) and Experiment 3 (**Figure 3B**) where no conflict adaptation was observed, pupil dilation data showed a post-conflict reduction of pupil dilation, which we propose to be a possible physiological marker of mental overload (cf. Granholm et al., 1996). However, the size of this effect did not scale with the behavioral measure of reduced conflict adaptation, so more research is warranted and future studies should consider to combine pupil dilation with additional measures, such as cardiovascular measures, that may provide a superior index of effort mobilization (Richter et al., 2008).

Although we have provided some initial evidence for an inverted U-shape between task difficulty and conflict adaptation, our work also shows how challenging it can be to establish the right experimental conditions under which these effects can be reliably investigated. In an ideal case, one would like to manipulate perceived task difficulty without introducing confounds in terms of overall speed and accuracy while at the same time keeping levels of potential motivation stable. Previous studies have aimed to do so by changing the appraisal of demands, e.g., using mood induction (Gendolla, 2000; van Steenbergen et al., 2010, 2012; cf. van Steenbergen, 2015). However, when introducing high task demands experimentally, participants can easily change their performance standards temporarily which might induce shifts in speed-accuracy that are difficult to assess at an individual level. When experimental conditions discourage such adaptation (as in our Experiment 3), this might in turn lower the potential motivation instead of increasing task difficulty, also resulting in reduced adaptation effects (see **Figure 5**).

Future work is also needed to understand the neural structures that track perceived task difficulty, and how these structures interact with brain areas involved in conflict monitoring (Botvinick et al., 2001). For example, we have recently proposed that rostral parts of the anterior cingulate cortex are involved in the evaluation (appraisal) of task difficulty, at least in the context of affect induction (van Steenbergen et al., 2015a). From a broader perspective, it is interesting to investigate how conflict adaptation relates to well-documented demand-driven improvements and impairments observed in more broadly defined types of cognitive- and self-control processes (Robinson et al., 2010; Koole et al., 2012; Proulx et al., 2012), and whether they reflect physical limitations in available resources or motivational limitations (Inzlicht et al., 2014b) that possibly could be counteracted by performance-contingent reward (Boksem and Tops, 2008; Sturmer et al., 2011; Braem et al., 2012; Tops et al., 2015).

An important take-home message of this study is also that highly demanding conflict tasks may not always produce the congruency-sequence effect as commonly reported in the literature. Our observations may also start to explain why some researchers using difficult tasks (e.g., using high incongruent-to-congruent ratios) have failed to observe typical conflict-adaptation effects (e.g., Wendt et al., 2007). Moreover, our work contributes to the cumulating evidence that various factors like trial pacing (Notebaert et al., 2006; Egner et al., 2010), time on task (Mayr and Awh, 2009; van Steenbergen et al., 2015b), incongruent-congruent ratio (Purmann et al., 2009; Duthoo et al., 2014), attempts to experimentally correct for feature binding effects (Akçay and Hazeltine, 2007; Duthoo et al., 2014), and type of conflict involved (Verbruggen et al., 2006) all may influence the size of congruency-sequence effects. Given that all these factors are likely to change task difficulty and motivation, it is an important aim for future research to better understand the commonalities of the mechanisms underlying effort mobilization and cognitive control and to develop models that are grounded in motivational accounts of action control (Shenhav et al., 2013; Inzlicht et al., 2014a; van Steenbergen, 2015).

## Conflict of Interest Statement

The authors declare that the research was conducted in the absence of any commercial or financial relationships that could be construed as a potential conflict of interest.

## Footnotes

1 Baseline pupil diameter was observed to be lower for the Stroop task (3.8 mm) in comparison to the flanker task (4.0 mm), *F*(1,21) = 54.0, *p* < 0.001. A separate analysis on pupil data from 13 participants who passively viewed 40 alternating 4-s baseline stimuli before the experiment started showed that this smaller pupil diameter in response to the Stroop baseline stimuli was likely due to luminance differences [3.68 versus 3.86 mm; *t*(12) = 6.5, *p* < 0.001]. These overall differences between pupil diameter thus did not allow for comparison of baseline pupil diameter between tasks (e.g., as an index of general arousal state). Task-evoked pupillary responses, however, are not likely to be affected by these differences because dilation measures were corrected for absolute diameter differences using baseline subtraction.
